# WI-TMLEGA: Weight Initialization and Training Method Based on Entropy Gain and Learning Rate Adjustment

**DOI:** 10.3390/e26080614

**Published:** 2024-07-23

**Authors:** Hongchuan Tang, Zhongguo Li, Qi Wang, Wenbin Fan

**Affiliations:** 1 School of Mechanical Engineering, Jiangsu University of Science and Technology, Zhenjiang 212100, China; tanghc@stu.just.edu.cn (H.T.); wqi003@126.com (Q.W.); 2 School of Automotive Engineering, Nantong Institute of Technology, Nantong 226001, China; 3 Jiangsu JBPV Intelligent Equipment Co., Ltd., Zhangjiagang 215634, China; fanwenbin@jbpv.com

**Keywords:** multilayer perceptron, MNIST dataset, weight initialization, weight update, learning rate

## Abstract

Addressing the issues of prolonged training times and low recognition rates in large model applications, this paper proposes a weight training method based on entropy gain for weight initialization and dynamic adjustment of the learning rate using the multilayer perceptron (MLP) model as an example. Initially, entropy gain was used to replace random initial values for weight initialization. Subsequently, an incremental learning rate strategy was employed for weight updates. The model was trained and validated using the MNIST handwritten digit dataset. The experimental results showed that, compared to random initialization, the proposed initialization method improves training effectiveness by 39.8% and increases the maximum recognition accuracy by 8.9%, demonstrating the feasibility of this method in large model applications.

## 1. Introduction

Large models generally refer to machine learning models with numerous parameters and complex structures, playing a significant role in fields such as natural language processing, image recognition, and speech recognition [1]. However, the complex structure of large models results in long training times and high computational power requirements.

In the field of large model training, weight initialization methods have a decisive impact on the learning ability and convergence speed of models [2]. In recent years, related research has mainly focused on analyzing how different initialization methods affect network convergence. For example, in 2021, Q. Yang proposed an adaptive covariance scaling estimation of distribution algorithm (ACSEDA) based on the Gaussian distribution model, which dynamically adjusts the variance of weights based on the characteristics of each layer (such as activation function type and connection density). By analyzing the depth and width of the network architecture, it automatically calculates the optimal initial variance for each layer to maintain healthy and stable gradients during training [3]. D. Johnson’s 2021 study explored a data-dependent weight initialization strategy, which customizes the initial distribution of weights by analyzing the mean and standard deviation of the dataset to ensure that the initialization state matches the data distribution [4]. R. Morales’ 2022 study combined the pretraining characteristics of deep belief networks to initialize weights. This method first trains the deep belief network in an unsupervised manner to capture and encode deep features of the input data, and then uses these features as initial weights for subsequent supervised learning tasks, improving the model’s performance on specific tasks [5]. In 2022, A. Tang proposed a regularization-adaptive initialization strategy, which optimizes the gradient flow during training by adjusting the variance of weights to adapt to different layers of the network, effectively reducing the occurrence of gradient vanishing at the early stage of training [6]. In 2023, A. Lopez proposed a robust initialization method based on gradient variance analysis, which adjusts the initial distribution of weights by analyzing the gradient variance of each layer at the initialization stage. This strategy aims to ensure more uniform gradient propagation between layers during training, reducing gradient vanishing and explosion issues, and enhancing training efficiency and model performance [7]. M. Magris’s 2023 survey proposed a Bayesian optimization-based neural network weight initialization method. This method employs Bayesian optimization techniques at the initialization stage, automatically searching for optimal weight initialization parameters based on prior knowledge and sample data. Specific steps include building a surrogate model based on Gaussian processes and using the acquisition function in the Bayesian optimization framework to guide the search for weight initialization parameters. This method effectively avoids gradient vanishing and explosion phenomena, improving the model’s convergence speed and accuracy [8]. E. Wilson’s 2023 study proposed a hierarchical progressive initialization method to enhance training stability by initializing network weights layer by layer. Specific steps include first initializing the weights of the bottom layer and performing short-term training, and then freezing the bottom layer weights and initializing the weights of the next layer, repeating this process until the top layer. After each layer is initialized, short-term training is performed to ensure normal gradient flow, optimizing the overall weight distribution of the network through a progressive layer-by-layer approach [9]. C. Nguyen’s 2023 study proposed a variance-preserving initialization method specifically designed for deep convolutional neural networks. This method calculates the variance of each layer’s input feature map during initialization and initializes weights to maintain equal variance between the input and output feature maps, preventing gradient vanishing and explosion. The implementation includes standardizing the convolution kernels of each layer and dynamically adjusting the distribution of initial weights [10]. M. Lee’s 2023 study proposed a meta-learning-based weight initialization method specifically for few-shot learning tasks. This method trains a meta-model during the meta-learning stage to learn how to quickly adapt to new tasks based on a small number of samples. In the specific implementation, a large-scale dataset is first used to pretrain the meta-model, and then the weights generated by the meta-model are used to initialize the network in few-shot learning tasks, improving the model’s adaptability and convergence speed on new tasks [11]. Some of these methods can accelerate convergence speed, and some can improve the stability of model performance, but there is still room for optimization.

In feature selection, information entropy can be used to evaluate the contribution of features to classification. The larger the information entropy, the greater the reduction in the uncertainty of the dataset after using the feature for division [12]. It has been proven that information entropy gain can be applied to optimize the structure of neural networks during training. For example, J. Carter and H. Nguyen’s 2021 study proposed a gene expression data analysis method based on information entropy gain, focusing on how to evaluate the contribution of different gene expressions to disease states using information entropy gain, thereby selecting the most influential genes as biomarkers. The method’s effectiveness was validated using actual clinical data, providing strong bioinformatics support for the early diagnosis and treatment of diseases [13]. In 2022, S. Richards and E. Thompson developed a new network intrusion detection system based on information entropy gain to evaluate abnormal patterns in network traffic. By calculating the information entropy gain of each data packet, the system can identify potential malicious traffic in real time while reducing false positives. The system was tested on multiple real-world datasets, demonstrating superior detection efficiency and accuracy compared to traditional methods [14]. A. Kumar and B. Lee’s 2021 study explored the application of information entropy gain in personalized recommendation systems. This method predicts new products or services that users may be interested in by analyzing the information entropy gain of users’ historical behavior. The model improved the scalability and efficiency of the recommendation system [15]. G. Zhao and Y. Wang’s 2022 study optimized a speech recognition algorithm using information entropy gain. By calculating the entropy value of speech signals, the study adjusted and optimized the parameters of the acoustic model. This method not only improved the accuracy of speech recognition, but also effectively reduced recognition errors in noisy environments [16].

During random weight initialization, the correlation coefficient between the information entropy gains of input features calculated during training and the corresponding neuron weights increases. Therefore, this study attempts to use information entropy for neural network weight initialization research.

The learning rate determines the step size of weight updates, directly affecting the training accuracy of neural networks. Therefore, the adjustment of the learning rate should also be dynamically conducted based on the actual training process. An appropriate learning rate can ensure rapid convergence of the network. In recent years, the study of step size has attracted much attention. For example, in 2021, F. Yang and C. Li proposed an adaptive learning rate adjustment strategy based on gradient changes. This strategy dynamically adjusts the learning rate by monitoring the gradient changes of model weights in real time. This method significantly improves training stability and model performance without sacrificing training speed [17]. In 2022, L. Zhang and K. Sharma studied a periodic learning rate adjustment method called Cyclical Learning Rate (CLR). By periodically adjusting the learning rate between a minimum and maximum value, this method aims to avoid local minima in the early stages of training and finely adjust model parameters in the later stages. Research shows that the CLR strategy improves model convergence speed and accuracy in multiple tasks, such as image recognition and language processing [18]. In 2021, M. Roberts and J. Turner developed a learning rate adjustment method combined with momentum factors. Their strategy not only considers the current gradient information, but also the cumulative impact of historical gradients, dynamically adjusting the learning rate and momentum parameters to optimize the training process [19]. In 2022, S. Gupta and A. Kumar proposed a simulated annealing-inspired learning rate adjustment strategy. By gradually reducing the learning rate, this method allows the model to explore the parameter space quickly in the early stages and find the global optimal solution more precisely in the later stages. This method is particularly suitable for training deep networks, effectively avoiding premature convergence to local minima [20]. This paper proposes a weight update method with an increasing learning rate strategy and compares it with some other methods.

The main contributions of this paper are summarized as follows:

- We propose an initialization method that uses information entropy gain instead of random weight initialization, enabling the model to better find the optimal weights during training, thereby reducing training time.
- During the weight update process, we employ a method of weight updating with an increasing learning rate strategy, allowing the model to complete training at the optimal learning rate, thereby improving recognition accuracy.

The relevant abbreviations for this paper are shown in Table 1.

## 2. Research Content

### 2.1. Introduction to Information Entropy Gain

Information entropy is used to describe the uncertainty or the amount of information in a random variable. In recent studies on large models, some researchers have utilized information entropy to measure the contribution of features to classification tasks in decision tree modeling, thereby enhancing model performance [21]. The formula for calculating information entropy is as follows:

(1) $$\begin{array}{c} H(D)=-\sum_{i=1}^{n}P(d_{i})\cdot {log}_{2}(P(d_{i})) \end{array}$$

where H(D) represents the information entropy of dataset D; $P(d_{i})$ denotes the probability of random variable d taking the value $d_{i}$, where d is a variable in dataset D; and n represents the number of values that random variable d can take.

Information entropy gain refers to an indicator used in feature selection to measure the impact of features on the overall uncertainty of the dataset. The calculation formula for information entropy gain is as follows:

(2) HG(D,A)=H(D)−H(D|A)

where HG(D,A) represents the information entropy gain of using feature A for classification under the condition of dataset D; H(D) denotes the information entropy of dataset D with multiple categories; H(D|A) represents the conditional information entropy of dataset D given the feature A.

Below is an example of calculating information entropy to illustrate the process of calculating information entropy gain. Suppose there is a dataset containing two features: A1 (age) and A2 (income), as well as a target variable D, whether to purchase. First, calculate the information entropy of dataset D; then, calculate the conditional information entropy of purchase behavior D given the A1; finally, compute the information entropy gain of age and income features. The calculation process is as follows:

Calculate the information entropy of the dataset H(D): Assuming purchase is positive, and non-purchase is negative, there are 60 instances of positive cases and 40 instances of negative cases in the dataset.

(3) $$H(D)=-\left(\frac{60}{100}\cdot {log}_{2}\left(\frac{60}{100}\right)+\frac{40}{100}\cdot \log_{2}\left(\frac{40}{100}\right)\right)\approx 0.971$$

that is, the overall information entropy H(D) of dataset D is approximately 0.971.

Calculate the conditional information entropy H(Age) of purchase behavior given the A1: Assuming there are three values for the age feature: youth, middle-aged, and elderly (with proportions of 30%, 40%, and 30%, respectively), for each age group, calculate the information entropy of purchase behavior, and then compute the information entropy based on the proportion of age:

(4) $$\begin{array}{c} H(D|Age)=\sum_{i=1}^{3}P(Age_{i})\cdot H(Age_{i}) \end{array}$$

For youth, middle-aged, and elderly, the specific numbers of samples for purchase and non-purchase are assumed as follows:

- Youth: Purchase 20, Non-purchase 10;
- Middle-aged: Purchase 30, Non-purchase 10;
- Elderly: Purchase 10, Non-purchase 20.

Based on these data, we can calculate the information entropy for each age group, and then combine their proportions to compute H(D|Age):

(5) $$H(D|Age)=\left(\frac{30}{100}\cdot H\left(D|Y\right)+\frac{40}{100}\cdot H\left(D|M\right)+\frac{30}{100}\cdot H\left(D|O\right)\right)\approx 0.0985$$

(6) HG(D,Age)=H(D)−H(Age)≈0.095

that is, the information entropy gain for the age feature on purchase behavior is approximately 0.095.

Suppose the income feature is divided into three levels: low, medium, and high (with proportions of 30%, 50%, and 20%, respectively), and the sample numbers are assumed as follows:

- Low income: Purchase 10, Non-purchase 20;
- Medium income: Purchase 30, Non-purchase 20;
- High income: Purchase 20, Non-purchase 0.

Similarly, the information entropy gain for the income feature on purchase behavior is approximately 0.21.

From the results, it can be observed that the information entropy gain of the income feature is higher than that of the age feature. This indicates that in the trained neural network model, the income feature should have greater weight.

### 2.2. Weight Initialization Methods

In the field of deep learning, neural network weight initialization can be carried out in various ways, such as Gaussian (normal) distribution initialization [22], uniform distribution initialization [23], truncated Gaussian distribution initialization [24], and principal component shuffling initialization [25], among others. Among these methods, popular weight initialization techniques such as Xavier initialization and He initialization [26] have been developed based on Gaussian and uniform distributions.

The Xavier initialization method uses a uniform distribution for weight initialization, following the main principle of maintaining consistency between the variance of activation values in forward propagation and the variance of gradient values of layer states in backward propagation during the propagation process. This enhances the smoothness of information propagation between network layers, thereby improving the efficiency and stability of network training.

The He initialization method, on the other hand, is an improvement based on Gaussian and uniform distributions, particularly suitable for layers using the ReLU activation function [27]. He initialization takes into account the characteristics of the ReLU activation function and adjusts the standard deviation of weights to adapt to the non-linear properties of the ReLU function in the positive interval, thus better supporting the training of deep learning networks.

However, due to the large difference between initial values and values at the end of training, networks using these two weight initialization methods still face the problem of long training times under the same update strategy. This paper intends to study the use of information entropy gain instead of random weight initialization and plans to compare it with the aforementioned initialization methods.

### 2.3. Current Learning Rate Adjustment Strategies

There are many learning rate adjustment strategies available [28], with the most common being learning rate decay [29]. The calculation formula is as follows:

(7) $$\begin{array}{c} \alpha_{i}=\alpha_{0}\cdot \frac{1}{1+d\cdot i} \end{array}$$

where i is the current iteration number, $\alpha_{i}$ is the learning rate at the i-th iteration, $\alpha_{0}$ is the initial learning rate, and d is the decay rate.

Subsequently, there emerged the method of periodic restarts of learning rates. The basic idea is to periodically adjust the size of the learning rate during training based on the iteration number. By resetting the learning rate to its initial value at the end of each iteration, it increases the model’s exploratory ability and convergence speed during training. For example, the Cyclical Learning Rate (CLR) [30] is calculated as follows:

(8) $$\begin{array}{c} \alpha_{i}=\alpha_{0}\cdot cos\left(\frac{\pi \cdot i}{T}\right) \end{array}$$

where $\alpha_{i}$ is the learning rate at the i-th iteration, $\alpha_{0}$ is the initial learning rate, T is the length of the cycle, which is the total number of iterations, and i is the current iteration number, thus utilizing the periodicity characteristics of the cosine function to adjust the learning rate.

However, the model still requires multiple cycles to find the optimal learning rate. To address this issue, this study added a learning rate increasing adjustment strategy to the model and compared it with the above two methods in terms of training accuracy. That is, the learning rate was dynamically increased and adjusted step by step during the training cycles of the neural network, and the learning rate value corresponding to the highest model accuracy state after all iterations are terminated were selected and saved for subsequent direct use.

## 3. Research Method

### 3.1. Model Construction

**Figure 1:**
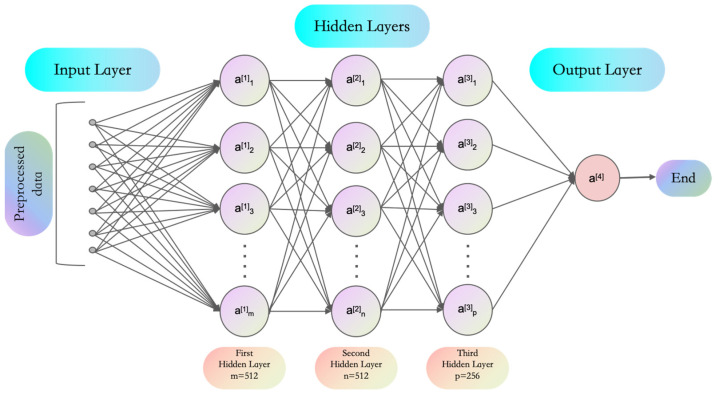
Model structure diagram illustrating the MLP network structure used.

The multi-layer perceptron (MLP) serves as a feedforward neural network primarily used for handling simple to moderately complex data problems [31]. The MLP model structure used in this study is shown in Figure 1: it includes an input layer, the first and second hidden layers with 512 neurons each, the third hidden layer with 256 neurons, and an output layer with 10 neurons. The model was trained with 128 batches and 12 epochs, with a total network parameter count of 398,186. This structure is suitable for training and testing as a large-scale simulation, with a simple weight initialization procedure facilitating result comparison and analysis.

### 3.2. Initialize Weights Using Normalized Information Entropy Gain

To facilitate comparison with the random initialization method, the information entropy gain values are normalized to (0, 0.001).

(9) $$\begin{array}{c} HN=\frac{{HG}_{r}-{HG}_{min}}{\left({HG}_{max}-{HG}_{min}\right)1000} \end{array}$$

where HN is the normalized information entropy gain, ${HG}_{r}$ is the information entropy gain of the r-th input feature, ${HG}_{min}$ is the minimum information entropy gain among all input features, and ${HG}_{max}$ is the maximum information entropy gain among all input features.

This method only changes the weight initialization from the input layer to the first hidden layer during the weight initialization process of the neural network; the weights of other layers are still randomly initialized.

**Figure 2:**
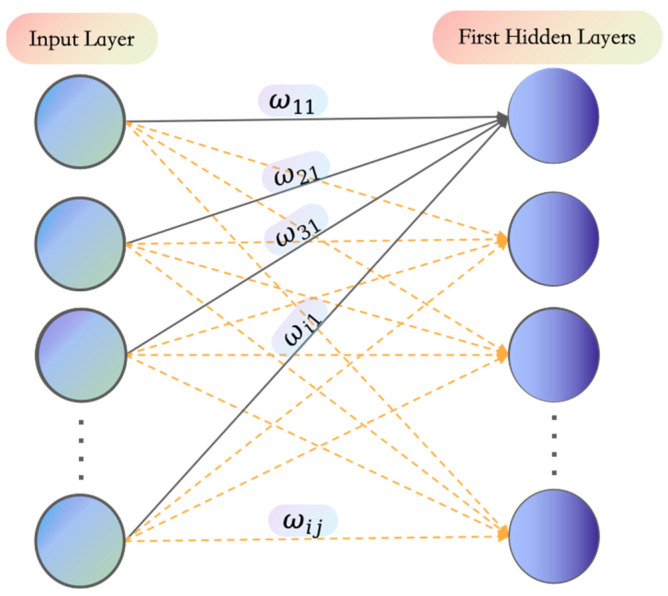
Schematic diagram of information displaying the specific locations of weights in the network connections.

As shown in Figure 2, the normalized information entropy gain $HN_{ij}$ is used as the initial value of the weight connecting the i-th input feature to the j-th neuron in the first hidden layer, that is:

(10) $$\omega_{ij}=HN_{ij}$$

where $\omega_{ij}$ is the weight corresponding to the i-th input feature and the j-th neuron (i∈ [1~*n*], *n* is the number of input features, and j∈ [1~512]).

### 3.3. Learning Rate Increment Strategy

To further optimize the training process of the model, this study introduces a learning rate increment adjustment strategy. This strategy ensures that as the number of iterations increases, the increment in the learning rate gradually decreases. Specifically, by setting an initial learning rate $\alpha_{0}$, the model is given a starting value, and the learning rate is incremented after each iteration according to a formula. Upon reaching the maximum number of iterations or the training error, the optimal learning rate is selected and saved, thereby improving the training efficiency and accuracy of the model. That is, when obtaining the learning rate $\alpha_{i}$ for the current iteration (i∈ [1~*N*], *N* is the total number of iterations), use:

(11) $$\alpha_{i}=\alpha_{i-1}(1+(\frac{\alpha_{i-1}}{\alpha_{0}}{)}^{\frac{1}{i}})$$

where $\alpha_{i-1}$ represents the learning rate from the previous iteration, and i represents the current iteration number. At the end of each training iteration, the next iteration’s learning rate $\alpha_{i}$ is computed by multiplying the current iteration’s learning rate $\alpha_{i-1}$ with a proportionality constant.

As indicated by Equation (11), when the maximum number of iterations *N* increases indefinitely, the coefficient multiplied by $\alpha_{i-1}$ in Equation (11) will approach 1, meaning that the growth rate of the learning rate will approach zero. Before reaching this state, the desired learning rate effect will have already been achieved. Therefore, this method can still function effectively even when the number of iterations is not fixed.

**Figure 3:**
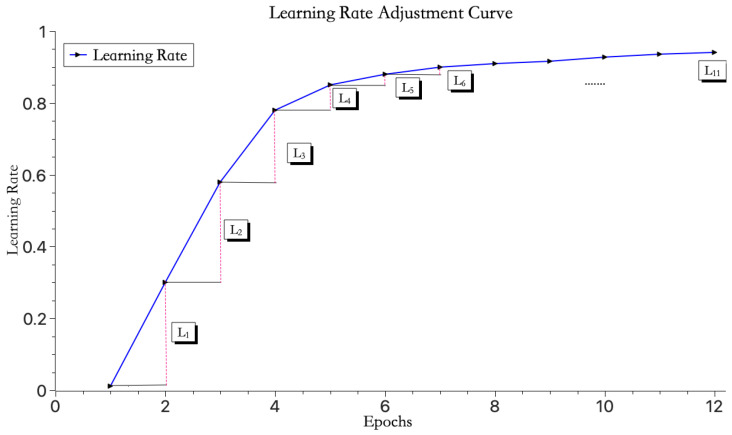
Learning rate change curve.

As shown in Figure 3, the learning rate initially increases rapidly and then gradually slows down with the increase in iteration count. Here, $L_{1}>L_{2}>L_{3}>L_{4}>L_{5}>L_{6}>\ldots >L_{11}$. This strategy helps the model quickly reach the ideal learning rate without adjusting too drastically and missing the optimal learning rate.

## 4. Experimental Results and Analysis

### 4.1. Experimental Running Environment and Number of Experimental Runs

The experiments were conducted on a Mac mini equipped with an Apple M2 chip, which has a total of 10 cores (4 performance and 4 efficiency cores), system firmware version iBoot-10151.1.1, and 8 GB of RAM. The operating system used was macOS, and the Python version was 3.10.9. Key libraries and frameworks included TensorFlow 2.9.0, scikit-learn 1.0.2, NumPy 1.22.3, and pandas 1.4.2.

All related experiments in this paper were conducted under the specified conditions and repeated 13 times to ensure the authenticity of the data and to verify the model’s stability.

### 4.2. Dataset Preprocessing

The model used in this study was trained and tested on the MNIST [32] handwritten digit dataset. This dataset contains a large number of handwritten digit images, each labeled with the corresponding digit. Each image has a pixel size of 28 × 28. The training set consists of 60,000 samples, while the test set includes 10,000 samples, covering handwritten digits from 0 to 9, each with a pixel size of 28 × 28. All experimental results in this paper are based on the test set.

**Figure 4:**
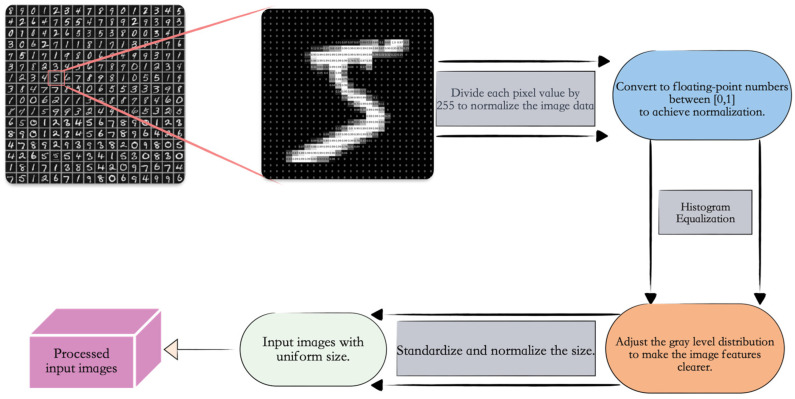
Dataset preprocessing flowchart showing the preprocessing workflow applied to the dataset used in this study.

First, the MNIST handwritten digit dataset was loaded, and each pixel value of the images was divided by 255 to convert the image data type to a more efficiently processed floating-point type, achieving normalization. This helps the model converge faster and improves its performance. Second, histogram equalization was applied to adjust the grayscale distribution of the images, making the grayscale distribution of the output images more uniform, thereby extracting clearer image features. Finally, the images were standardized in size, adjusting all images to the same dimensions to ensure uniform input sizes for the neural network, as shown in Figure 4.

### 4.3. Convergence Speed

The reason for discussing and studying the speed of weight convergence in this paper is that when the model’s weights converge quickly, it indicates that the model achieves the expected performance in fewer iterations. This implies that the model finds a better parameter combination in a shorter time, thereby reducing the overall training time. Therefore, this paper uses the speed of weight convergence as a proxy for measuring the extent of training time reduction. During the experiments, irrelevant variables (such as data size and model complexity) were controlled to remain constant, while only the weight initialization methods were varied. This approach aims to establish the relationship between weight initialization and its impact on model performance. The specific experimental process is as follows.

Due to the significant variation in the weights between the same input variable and different neurons in the hidden layer during training, for ease of subsequent comparison, the weights of the neurons in the first hidden layer connected to the same input variable are averaged. The calculation formula is as follows:

(12) $$\omega_{i1}=\frac{\sum_{b=1}^{512}\omega_{ib}}{512}$$

where $\omega_{i1}$ is the average weight of all 512 neurons in the first hidden layer connected to the i-th feature, and $\omega_{ib}$ is the weight of each neuron in the first hidden layer connected to the i-th feature (*b* ∈ [1~512]).

**Figure 5:**
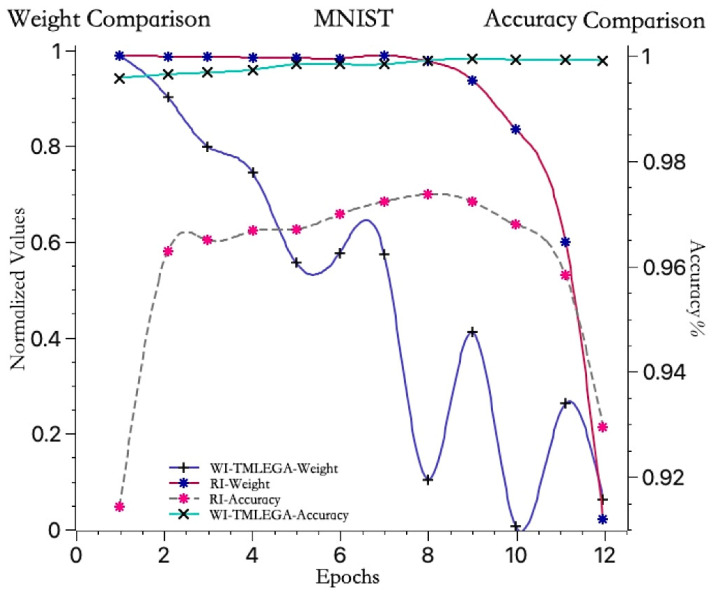
Comparison of convergence speed before and after weight initialization improvement. The blue and red weight convergence curves correspond to the left vertical axis, while the light blue and gray accuracy curves correspond to the right vertical axis. The maximum accuracy value indicates that the model has found the optimal weights for the current iteration.

The convergence trends of the average weights for the model initialized with information entropy gain and the model initialized with random weights, as well as the accuracy trends of the information entropy gain-initialized model and the random-initialized model, are shown in Figure 5.

The red curve represents the convergence trend of the average weight of the first hidden layer in the model initialized with random weights. It remains relatively stable before the 8th iteration, with a small decrease, hovering around 1, and then sharply drops to near 0 between the 9th and 12th iterations. On the other hand, the blue curve represents the convergence trend of the average weight of the first hidden layer in the model initialized with information entropy gain. It shows an approximate downward trend before the 6th iteration, decreasing from 1 to 0.6, and then rapidly dropping to around 0.1 at the 8th iteration, with some fluctuations from the 9th to 12th iterations, ultimately approaching 0. Throughout the entire process, it can be observed that the value of the blue curve remains consistently lower than that of the red curve. This indicates that the weight convergence speed of the WI-TMLEGA method is faster than that of the RI method, suggesting that adopting the WI-TMLEGA method reduces the distance to the optimal solution and, thus, approaches the optimal solution more rapidly.

The gray dashed line represents the accuracy curve of the model initialized with random weights. It remains relatively stable before the 8th iteration and reaches its maximum accuracy at the 8th iteration, followed by a rapid decline in accuracy from the 9th to 12th iterations. Meanwhile, the light blue curve represents the accuracy curve of the model initialized with information entropy gain. It shows relatively small fluctuations and a stable upward trend, starting with the lowest accuracy in the first iteration, but still far above the maximum accuracy of the randomly initialized model. The analysis indicates that the accuracy improvement of the model initialized with information entropy gain is more stable, indicating the good generalization ability of the model.

**Figure 6:**
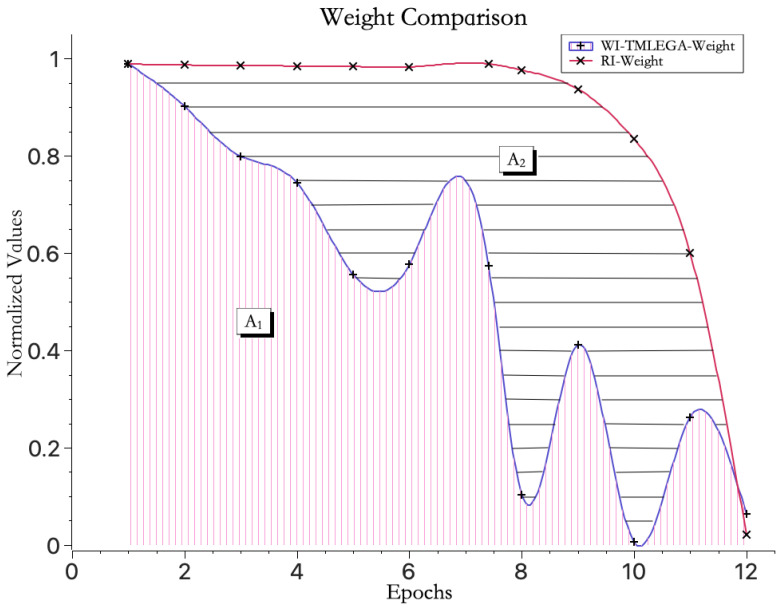
Area under the weight convergence speed curve. “$A_{1}$” represents the area enclosed by the weight mean convergence curve and the horizontal axis when using the WI-TMLEGA method for weight initialization. “$A_{2}$” represents the area enclosed by the weight mean convergence curve using the RI method for weight initialization and the curve using the WI-TMLEGA method, which indicates the improved convergence efficiency provided by the WI-TMLEGA method.

From a calculus perspective, the area under the curve represents the integral value of weight convergence, and the smaller the area under the curve, the faster the convergence speed. Therefore, as shown in Figure 6, when both curves before and after improvement are displayed on the same axis, the area $A_{1}$ enclosed by the WI-TMLEGA curve and the x-axis, and the area $A_{2}$ enclosed by the WI-TMLEGA curve and the RI curve, further demonstrate the significant advantage of the improved model in weight convergence speed. The improvement in convergence speed T compared to the original random initialization method can be obtained according to the following formula:

(13) $$T=(1-\frac{\int_{0}^{12}w_{1}dE}{\int_{0}^{12}w_{2}dE})\cdot 100\%$$

which means:

(14) $$T=(\frac{A_{2}}{A_{1}+A_{2}})\cdot 100\%$$

In this equation, T represents the percentage increase in convergence speed, $w_{1}$ denotes the convergence speed of the random initialization method, $w_{2}$ represents the convergence speed of this method, and E stands for the number of epochs on the x-axis.

The calculations show that T equals 39.8%.

Through the comprehensive analysis of the experimental results, it is evident that the improved WI-TMLEGA initialization model significantly accelerates the convergence speed of weights. This has a crucial impact on both the training efficiency and accuracy of the model, effectively enhancing the performance and practical value of the digit recognition model.

**Figure 7:**
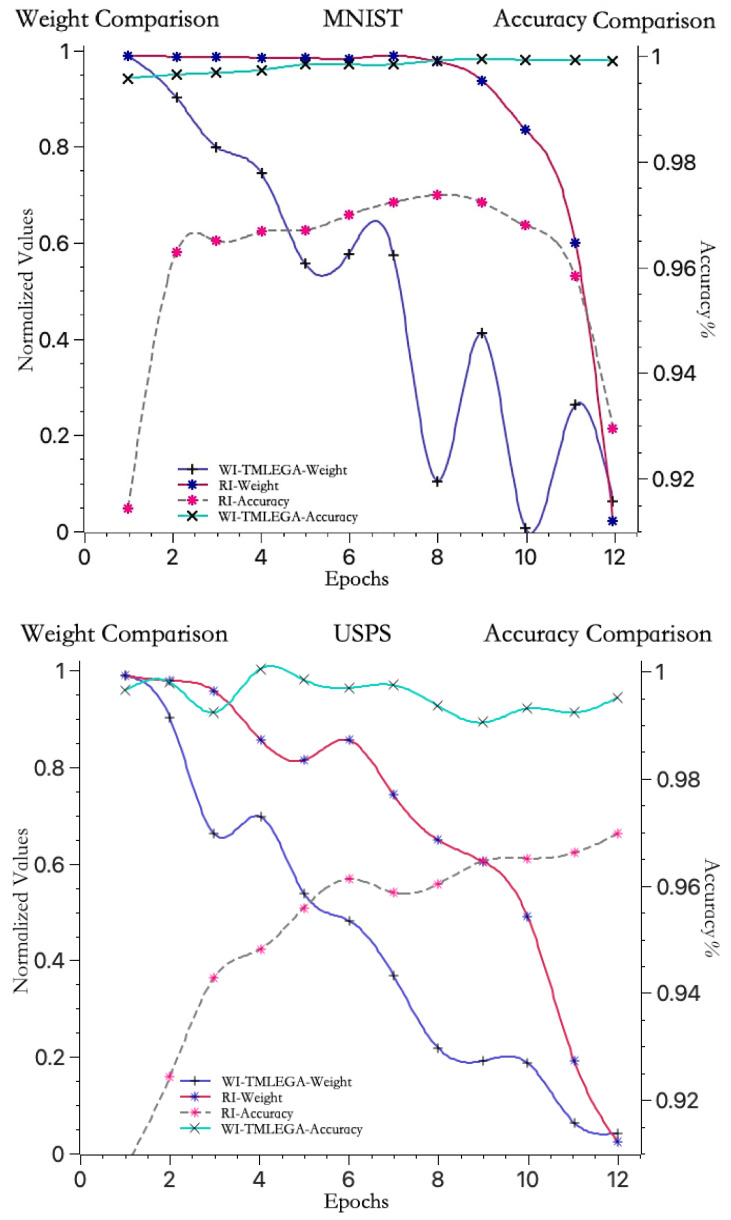

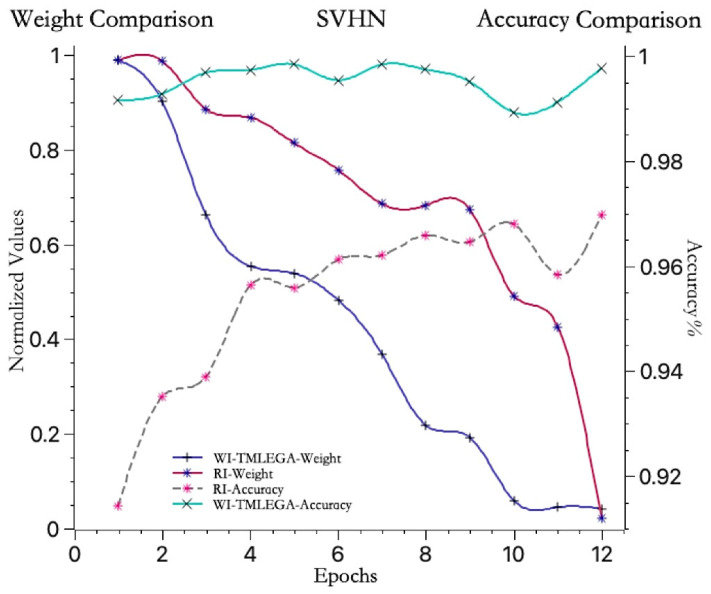
The WI-TMLEGA method’s performance comparison across different datasets is as follows: the first chart depicts the MNIST dataset used in this study; the second chart illustrates the USPS dataset, featuring handwritten digits primarily utilized for postal-service-related automatic recognition and classification tasks; and the third chart displays the SVHN dataset, comprising digit images extracted from Google Street View, with each image containing one or multiple digits, used as a benchmark for multi-digit classification and localization tasks.

As shown in Figure 7, to further clarify the performance of the WI-TMLEGA method on different datasets, this paper compares it with the MNIST dataset using the USPS [33] and SVHN [34] datasets. First, the USPS and SVHN datasets were obtained from public sources and processed as described in Section 4.2. The USPS and SVHN datasets were then divided into training and test sets according to the same proportions as the MNIST dataset. Subsequently, the models using the USPS and SVHN datasets were initialized with both RI and WI-TMLEGA methods, and their performance was evaluated. The model architecture, number of training epochs, and parameters remained unchanged. According to the results presented in Figure 7, the WI-TMLEGA method demonstrates varying degrees of performance improvement over the RI initialization method across different datasets.

### 4.4. Accuracy

#### 4.4.1. Different Initialization Methods

**Figure 8:**
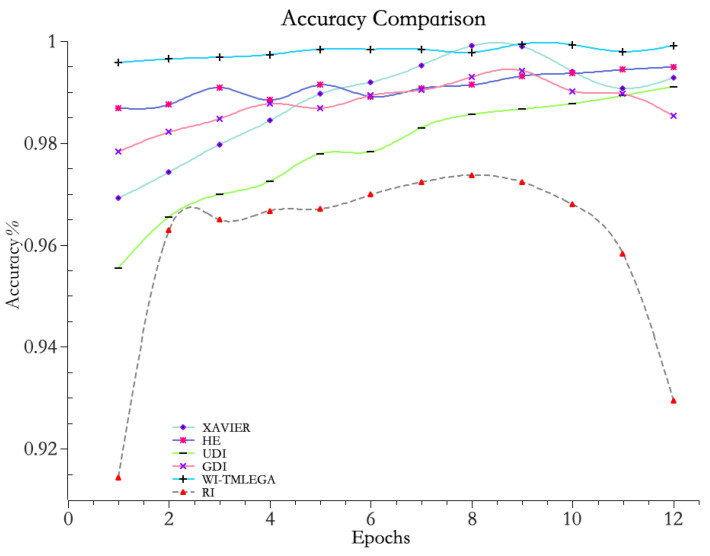
Comparison between WI-TMLEGA method and other initialization methods. The accuracy of the model was assessed using five common initialization methods. A larger value indicates higher accuracy.

Experimental comparisons were conducted between the WI-TMLEGA method and random initialization (RI), He initialization, Xavier initialization, Gaussian distribution initialization (GDI), and uniform distribution initialization (UDI) on the same dataset. The experimental results are shown in Figure 8 and Table 2.

From Figure 8, it is evident that the accuracy curve of the improved WI-TMLEGA method consistently remains above 99% and exhibits a relatively stable trend. This indicates that the model, after the improvement, achieves a very high level of prediction accuracy. In summary, the model improved using the WI-TMLEGA method shows superior performance in terms of accuracy and stability.

From the data in Table 2, it can be seen that when using the WI-TMLEGA method, the maximum difference in accuracy compared to the traditional RI method appears during the first iteration, with a peak accuracy difference of 0.08194. This indicates that the maximum improvement in model accuracy when applying this method is:

(15) $$\frac{0.08194}{0.91384}\times 100\%\approx 8.96\%$$

#### 4.4.2. Different Learning Rate Adjustment Strategies

**Figure 9:**
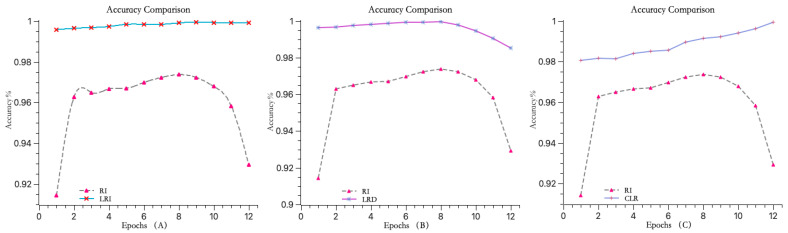
Comparison of three learning rate change strategies. (**A**) The accuracy curve of the model using a learning rate increment function steadily rises between 0.95 and 1. (**B**) The accuracy curve of the model using a learning rate decrement function gradually decreases to around 0.98 after the seventh iteration. (**C**) The accuracy curve of the model using a fixed learning rate function, although steadily increasing throughout, starts with an initial accuracy of only 0.98. Through a comparison using the same dataset, it is evident that the model using a learning rate increment function can effectively improve accuracy.

In this experiment, three different learning rate adjustment strategies were compared after optimizing the model using the WI-TMLEGA initialization method: increasing learning rate (as shown in Figure 9A), decreasing learning rate (as shown in Figure 9B), and constant learning rate (as shown in Figure 9C). The accuracy of the model was evaluated and compared, as shown in Table 3.

In Table 3, the GAP values indicate the extent of accuracy improvement when using the WI-TMLEGA initialization method with different learning rate adjustment strategies. A larger GAP value suggests a more pronounced improvement. According to the data in Table 3, in 12 iterations, the method using a learning rate increment strategy had the highest GAP value in seven instances and the second highest in five instances. Therefore, it is evident that models trained using the learning rate increment strategy achieve higher accuracy levels.

Based on the experimental results, it can be concluded that when training the model using an increasing learning rate strategy, the model’s accuracy gradually increases with the number of iterations during the first ten iterations. However, it decreases by 0.0004 during the 11th and 12th iterations. This indicates that the increasing learning rate strategy not only improves the model’s accuracy, but also ensures a certain degree of stability.

## 5. Conclusions

In this study, we proposed a neural network weight initialization method based on information entropy gain, called WI-TMLEGA, aimed at addressing the issue of long training times for large models. Firstly, using the normalized information entropy gain for neural network weight initialization can more effectively utilize the important information of features. This adjusts the range and distribution of weight initialization, giving higher initial values to the weights corresponding to important features. This improvement in the weight convergence rate reflects a reduction in training time. Secondly, we added an increasing learning rate strategy to help the model quickly reach the desired learning rate without adjusting too much and missing the optimal learning rate. Finally, using the MNIST handwritten digit dataset for model training and testing, the experimental results demonstrate significant improvements in training speed, model accuracy, and weight convergence speed compared to traditional random initialization methods.

These achievements highlight the potential and practical value of WI-TMLEGA in the field of optimizing deep learning neural network structures. Furthermore, the conclusions and methods derived from this study’s improvements to and optimization of MLP can be extended to other deep learning networks.

Future research directions may include exploring WI-TMLEGA’s application in more complex datasets (models) to verify its generalizability across various domains.

## 6. Patents

The work reported in this manuscript led to the filing of a patent, currently in the acceptance stage. The patent is entitled “A Method for Neural Network Weight Initialization and Training for Digital Recognition,” with the application number 202410091020.8. This patent encompasses an innovative approach that utilizes normalized information entropy gain to initialize neural network weights and incorporates a learning rate increment strategy. It aims to address some limitations of current large model technologies, improve the training accuracy of large models, and reduce training time. Through this patent application, this research seeks to advance the field of large models, particularly in the area of image recognition models.

## Figures and Tables

**Table 1:** List of acronyms used in this paper.

Acronym	Description
MLP	Multilayer perceptron
WI-TMLEGA	Initialization information entropy gain and learning rate adjustment
TM	Traditional methods
LRI	Learning rate increment
GAP	Discrepancy
LRD	Learning rate decrement
CLR	Learning rate constant
MAG	Maximum accuracy gap
RI	Random initialization
GDI	Gaussian distribution initialization
UDI	Uniform distribution initialization

**Table 2:** Accuracy comparison of 6 initialization methods on the same dataset. The specific values of model accuracy using different initialization methods on the same dataset are displayed. A larger value indicates a more significant improvement in model accuracy at the current iteration. The maximum value is highlighted in blue.

Epochs	RI	WI-TMLEGA	HE	XAVIER	GDI	UDI
1	0.91384	0.99578	0.98676	0.96883	0.97861	0.95539
2	0.96281	0.99654	0.98716	0.97413	0.98228	0.96537
3	0.96471	0.99673	0.99063	0.97963	0.98492	0.96985
4	0.96643	0.99749	0.98818	0.98431	0.98757	0.97230
5	0.96700	0.99845	0.99103	0.98981	0.98696	0.97780
6	0.96948	0.99845	0.98920	0.99185	0.98900	0.97800
7	0.97215	0.99845	0.99042	0.99490	0.99042	0.98268
8	0.97368	0.99902	0.99144	0.99938	0.99287	0.98553
9	0.97177	0.9994	0.99287	0.99892	0.99409	0.98655
10	0.96758	0.9994	0.99348	0.99893	0.99022	0.98798
11	0.95804	0.9994	0.99429	0.99022	0.98981	0.98900
12	0.9291	0.99902	0.99470	0.99226	0.98553	0.99103

**Table 3:** Impact of different learning rate change strategies under WI-TMLEGA initialization. Specific data on the impact of different learning rate adjustment strategies on model accuracy in comparative testing: the symbol ↑ represents the highest accuracy among the three strategies at the current iteration, and GAP stands for the difference in accuracy compared to the RI method, with a larger difference indicating a better improvement in accuracy. The improvement in accuracy is highlighted in red, while the second-best results are highlighted in green.

Epochs	Learning Rate Increment			Learning Rate Decrement			Constant Learning Rate		
	TM	LRI	GAP	TM	LRD	GAP	TM	CLR	GAP
1	0.9141	0.9969	0.0828	0.9141	0.9970↑	0.0829	0.9141	0.9816	0.0675
2	0.9633	0.9977↑	0.0344	0.9633	0.9974	0.0341	0.9633	0.9824	0.0191
3	0.9647	0.9974	0.0327	0.9647	0.9983↑	0.0336	0.9647	0.9824	0.0177
4	0.9675	0.9983	0.0308	0.9675	0.9987↑	0.0312	0.9675	0.9845	0.0170
5	0.9682	0.9987	0.0305	0.9682	0.9991↑	0.0309	0.9682	0.9854	0.0172
6	0.9696	0.9991	0.0295	0.9696	0.9995↑	0.0299	0.9696	0.9862	0.0166
7	0.9724	0.9996↑	0.0272	0.9724	0.9994	0.0270	0.9724	0.9899	0.0175
8	0.9745	0.9999↑	0.0254	0.9745	0.9995	0.0250	0.9745	0.9916	0.0171
9	0.9722	0.9999↑	0.0277	0.9722	0.9983	0.0261	0.9722	0.9929	0.0207
10	0.9673	0.9999↑	0.0326	0.9673	0.9949	0.0276	0.9673	0.9945	0.0272
11	0.9584	0.9995↑	0.0411	0.9584	0.9908	0.0324	0.9584	0.9962	0.0378
12	0.9296	0.9992↑	0.0696	0.9296	0.9858	0.0562	0.9296	0.9991	0.0695

## Data Availability

This manuscript encompasses all data that were produced or examined throughout the course of this study. Accompanying scripts and computational methods integral to the data’s creation will be made available in due course. The funding for this study was provided by the Jiangsu Key Research and Development Program.
